# Farther Observations on the Use of Opium, in Removing Symptoms Supposed to Be Owing to Morbid Irritability
*See page 5.


**Published:** 1785

**Authors:** 


IV. *Farther Obſervations on the Uſe of Opium, in removing Symptoms ſuppoſed to be owing to morbid Irritability*. Communicated in a Letter to Dr.* Simmons, *by Mr.* Alexander Grant, *Senior Surgeon of His Majeſty's Military Hoſpitals during the late War in North America.*

IN the winter of the ſame year † in which I employed Opium ſo ſucceſsfully in caſes,

* See page 5. † 1779.

originally originally venereal, and where mercury ſeemed to be of no efficacy, a number of patients were received into the General Hoſpital, at different times, with ſome of the extreme parts of the body mortified, in conſequence of moſt ſevere cold weather.

This ſpecies of mortification is a very common accident, or diſeaſe, in all cold climates, eſpecially among ſoldiers, whoſe duty expoſes them to the ſeverity of the weather.

I have often ſeen a perſon's noſe and ears, but particularly the latter, bliſtered from cold, at a time when his body has been in full exerciſe of either riding on horſeback, or walking. Some conſtitutions ſeem to feel the effects of cold ſooner, and in a more ſevere manner, than others.

The parts of the body that I have in general obſerved to be froſt nipped, are the fingers and toes, or the noſe and ears. When either of the two latter are affected, the conſequences are ſeldom very ſerious; but the former are ſeldom froſt-nipped, without the loſs of ſome of the joints.

Before the period above mentioned, I had had opportunities of meeting with many caſes of this ſort, and had generally adopted the ſame applications to the parts, and the ſame medicines internally, as are uſually recommended in caſes of ſphacelus or gangrene ; but having ſucceeded to my wiſhes in exhibiting Opium in caſes of morbid irritability, I was induced to extend my trials of that remedy to the ſpecies of mortification in queſtion. Accordingly, I began with giving it as ſoon after the attack as poſſible, and with the beſt effects, having, at no time, had occaſion to make uſe of any other medicines, whilſt any of the ſymptoms continued.

Theſe ſymptoms are, in general, moſt excruciating pain ; a quick, and in ſome conſtitutions, a ſtrong pulſe ; total want of reſt ; thirſt ; anxiety ; and, frequently, a nauſea.

Before I proceed to relate particular caſes, I think it right to obſerve, that as ſoon as the Opium was found to anſwer the intended purpoſes, by alleviating pain and diminiſhing the circulation, I omitted it, and completed the cure in the uſual manner. And here I may remark, that in caſes of this kind, as well as in ulcers in general, no uſeful change on the ſurface of the ſore will be produced till the pain, and other ſymptoms of morbid irritability, are leſſened.

When the irritability appears to be only local, the Thebaic ſolution *, by its action on the ſurface of the ſore, I have often ſeen produce ſingularly good effects in obſtinate caſes †. I have uſually applied it twice a day, and ſometimes oftener, in an oatmeal poultice, and have found this mode preferable to that of pledgits of lint dipped in the ſolution, as theſe ſoon become dry, and the lint then adheres to the ulcer, occaſions pain, and retards the relaxation of the ſore, which is the firſt neceſſary alteration that muſt take place; whereas the poultice, by continuing moiſt a longer time, obviates many difficulties, and allows the Opium to act. This method will, in general, ſoon produce an uſeful change, and afford an opportunity of uſing other applications with ſucceſs; though in ſome caſes this dreſſing will be proper to be continued till within a few days of the cure.

* See page 12.

† I do not recollect to have experienced the leaſt ill conſequence from the application of the Thebaic ſolution, of any degree of ſtrength, externally, by its affecting either the head or the bowels; and the pain that is now and then ſuppoſed to be occaſioned by it, ſeldom continues longer than the firſt dreſſing.

I ſhall now beg leave to add the three follow-ing caſes, (ſelected from a great number) in which the foundation of a cure was effected by uſing Opium internally.

In the firſt of theſe caſes may be obſerved the good effects of exhibiting it immediately after the accident; in the ſecond, I did not exhibit it till the end of two days; and in the third, not till after the uſual method of treating ſuch caſes had failed.

### C A S E I.

JOHN WOOD, about 23 years of age, was brought into the hoſpital, on the morning of the 30th of December, 1779, with both feet ſphacelated, in conſequence of the effects of cold. Upon inquiry, I found that he had fallen down, and had contuſed the whole of the right upper extremity, which occaſioned great ſwelling and inflammation, attended with much pain, a ſtrong full pulſe, nauſea, and frequent inclination to vomit. I adminiſtered immediately one grain of Opium; applied ſoft emollient poultices to the parts, and directed one grain more of Opium to be taken at night, and to be repeated the following morning.

On the 31ſt, the inclination to vomit was not ſo frequent, and he was diſpoſed to reſt. The doſe of Opium was ordered to be continued night and morning.

January 1ſt. The arm and both feet were much the ſame as before, but the vomiting was conſiderably better.

On the 2d, the vomiting ceaſed; he was in every other reſpect better, and there was an appearance of the ſlough's ſeparating.

The 3d, 4th, 5th, and 6th, as he was much the ſame, I increaſed the doſe of Opium at night to two grains,

On the 7th he was better.

And on the 9th he was in a good ſtate.

On the 11th, his arm was quite well, and both his feet as well as could be expected, as he complained of no pain, and enjoyed his reſt. Between the 11th and 14th, the ſloughs were thrown off from all the toes, except the little toe of the right foot; and he was in other reſpects going on ſo well, that I decreaſed the doſe of Opium at night one grain.

On the 20th, I removed the ſmall toe (which I found much more affected than any of the others, the ſlough being very deep) at the metatarſal joint. At this time, the ſwelling and inflaminflammation were quite reduced, the poultices were difcontinued, and the fore dreffed in the fimpleft manner.

On the 23d, I omitted the dofe of Opium in the morning, and on the 25th omitted it altogether. From this time to the 13th of March, when the right foot was quite healed, he was gradually doing well; and on the 22d, the left foot was likewife healed.

### CASE II.

JANUARY 3d, 1780, George Walker was brought into the hofpital, with a fphacelus of all the toes of both feet, extending to the metatarfus, in confequence of expofure to cold. He complained of great pain, and of want of reft. I directed the parts to be well fomented and poulticed, and the fame applications to be repeated at night, without any medicine. But on the 5th, as he was no better, I ordered him to take one grain of Opium night and morning.

On the 6th, he was rather eafier.

On the 7th and 8th, perceiving but little alteration, I increafed the dofe of Opium to two grains night and morning.

On the 9th, he was a little better, and the floughs were beginning to feparate.

On the 10th, he was going on well.

On the 11th, he complained of a numbnefs of his legs and thighs, but his pulfe was extremely good; the floughs continued to feparate till the 24th, when I took off the great toe of his right foot at the metatarfal joint; in every other refpect he was very well.

February 1ft, I omitted the dofe of Opium in the morning. The fores were now in a healing ftate.

On the 11th, I omitted the dofe of Opium altogether; and from the 20th of February till the 18th of March, when he was perfectly cured, the parts were gradually healing.

### C A S E III.

FEBRUARY 10th, 1780, I attended, with other gentlemen of the hofpital, John Hedder, about 51 years of age, who, from expofure to cold on the 25th of the preceding month, had a fphacelus of all the toes of both feet, and an evident appearance of its fpreading to the ancle. The inflammation and fwelling were very confiderable above the ancle; and at this time the pain was excruciating, and he was without reft.

From the 25th of January till the 10th of February, a variety of changes had happened, fometimes feemingly for the better, at other times for the worfe; and he had frequently complained of naufea, and of an irritation of the bowels, attended with a weak quick pulfe. The treatment had been varied according to the urgency of the fymptoms; fomentations, digeftives, and poultices, had been at different times applied, but as neither of thefe were productive of the ufual good effects, it was thought adivfeable to omit the whole of the plan he was then upon, which was accordingly done, and he was directed to take three grains of Opium directly, and two grains more at bed-time. Thefe dofes were ordered to be repeated every night and morning, and the parts to be dreffed with a foft emollient poultice only.

On the 11th, the pain was much relieved; his pulfe was a little ftronger, and nearer its natural ftate; and he had refted very well the laft night.

On the 12th, he had eafe and reft; his pulfe was good, and there was an appearance of the floughs feparating.

On

On the 13th he was a good deal better, and had a good ſuppuration, with eaſe and reſt.

On the 14th, the ſloughs were nearly looſe.

On the 15th, he continued in a good ſtate.

On the 16th, the ſeparation of the ſloughs leaving the bones bare, ſome of the toes were removed at the tarſal, and the others at the metatarſal joint.

On the 17th, as he was going on well, we began to leſſen the doſe of Opium; and on the 3d of March it was omitted altogether.

*St. Alban's Street,*

May 9th, 1785.

